# Depressive symptoms and associated factors among pregnant women attending antenatal care at Comprehensive Specialized Hospitals in Northwest Ethiopia, 2022: an institution-based cross-sectional study

**DOI:** 10.3389/fpsyt.2023.1148638

**Published:** 2023-06-21

**Authors:** Girmaw Medfu Takelle, Girum Nakie, Gidey Rtbey, Mamaru Melkam

**Affiliations:** Department of Psychiatry, College of Medicine and Health Science, University of Gondar, Gondar, Ethiopia

**Keywords:** depression, pregnant, women, Ethiopia, symptoms

## Abstract

**Background:**

Depression during pregnancy has a significant impact on public health as it can adversely affect both the mother's and the child's health. These can have devastating effects on the mother, the unborn child, and the entire family.

**Objective:**

This study aimed to determine the prevalence of depressive symptoms and associated factors among pregnant women in Ethiopia.

**Method:**

An institutional-based cross-sectional study was conducted among pregnant women attending antenatal care services at comprehensive specialized hospitals in Northwest Ethiopia from May to June 2022.

**Measurement:**

The desired data were collected through face-to-face interview techniques by using validated questionnaires such as the Edinburgh Postnatal Depression Scale, the Oslo-3 social support scale, and the Abuse Assessment Screen tools. The data were analyzed by using SPSS Version 25. Logistic regression analysis was used to identify factors associated with antenatal depressive symptoms. Variables having a *p*-value of <0.2 in the bivariate analysis were entered into the multivariable logistic regression. A *p*-value of <0.05 was considered statistically significant, at 95% CI.

**Results:**

This study revealed that 91 (19.2%) pregnant women screened positive for depressive symptoms. According to multivariable logistic regression, living in rural areas (adjusted odds ratio (AOR) = 2.58, 95% CI: 1.267, 5.256), being in the second or third trimesters of gestational phase (AOR = 4.40, 95% CI: 1.949, 9.966 and AOR = 5.42, 95% CI: 2.438, 12.028, respectively), having a history of alcohol use (AOR = 2.41, 95% CI: 1.099, 5.260), having moderate or poor social support (AOR = 2.55, 95% CI: 1.220, 5.338 and AOR = 2.41, 95% CI: 1.106, 5.268), and having a history of intimate partner violence (AOR = 2.67, 95% CI: 1.416, 5.016) were the factors significantly associated with depressive symptoms at a *p*-value of ≤ 0.05.

**Conclusion and recommendation:**

The prevalence of depressive symptoms among pregnant women was high. Living in rural areas, second and third trimesters, use of alcohol, having moderate to poor social support, and having a history of intimate partner violence were variables significantly associated with depressive symptoms during pregnancy.

## Introduction

Despite societal expectations that a woman's emotional wellbeing should be prioritized during pregnancy, changes in social roles and self-definition, along with other risk factors like poverty, repeated intimate partner violence or domestic violence, poor social support, food insecurity, unplanned pregnancy, and low access to education, might make pregnant women more susceptible to mental health problems in low- and mid-income countries (1–3).

Depression is one of the most common mental disorders that present with a depressed mood, loss of pleasure or interest, decrease in energy, a feeling of guilt or low self-worth, disturbed sleep and appetite, and poor concentration (4). Depression affects one in every five people and is the most common expressive disorder in women and the general population, with a more than 2-fold higher incidence in women than in men (5, 6). By 2020, depressive disorders were anticipated to be the second-leading contributor to the burden of disability worldwide, and by 2030, the condition is expected to take first place (7, 8).

One of the most common psychiatric conditions affecting women is antenatal depression (7, 9). Antenatal depressive disorder is a physical, mental, and emotional condition that lasts longer than 2 weeks and is characterized by persistent feelings of low mood and hopelessness as well as a lack of interest to engage in formerly enjoyable activities (5).

According to a number of studies, pregnant women experience antenatal depression more frequently than postpartum women do (5, 9–13).

Antenatal depression, also known as prenatal or perinatal depression, is a form of clinical depression that can affect a woman during pregnancy and can be a precursor to postpartum depression if not properly treated (14). Feelings of fatigue, tearfulness, helplessness, anxiety or impairment in attention and concentration, changes in appetite, insomnia, and suicidal thoughts are a few of the symptoms (15). According to studies, 25–35% of expectant mothers experience depressive symptoms, and 20% of them may experience serious depression at some point during the pregnancy (15, 16).

Antenatal depression can happen at any stage of pregnancy and can be a response to the pregnancy itself, brought on by health problems, significant life pressures, genetic and metabolic factors, or the continuation or recurrence of a pre-pregnancy illness (9, 17). Antenatal depression can have devastating effects on the mother, the unborn child, and the entire family (18). Premature birth, low birth weight, intrauterine growth restriction, postpartum depression, preeclampsia, anemia, learning difficulties, malnutrition and febrile illness, respiratory problems, and intellectual disability are all negative consequences of antenatal depressive symptoms (8, 18–22). Additionally, a high rate of peptide and steroid hormone fluctuation that occurs during pregnancy and childbearing age is likely to make antenatal depressive symptoms worse (8, 23, 24).

The prevalence of antenatal depression ranges from 7% in Taiwan and Australia (25–27) to 39.5% in Tanzania (28). In developed countries, a meta-analysis study found that the prevalence rates of depression were 7.4% (2.2–12.6%), 12.8% (10.7–14.8%), and 12.0% (7.4–16.7%) during the first, second, and third trimesters, respectively (10). A high prevalence of depressive symptoms or probable depression during pregnancy has been reported from developing countries, i.e., 18% in Bangladesh (29), 24.8% in Nepal (30), 25 % in Pakistan (22), 20.2 % in Brazil (19), 39 % in South Africa Cape Town (20), 38.5 % in South Africa KwaZulu-Natal (21), 31% in Limpopo Province South Africa (31), 39.5 % in Tanzania (28), 26.9% in Ghana (32), 29.4% in Nigeria (33), and 15.2 %−35.4% in Ethiopia (9, 34–36).

Previous studies showed that prior history of depression, unplanned pregnancy, lack of the baby's father, unemployment, current or previous exposure to different forms of abuse or violence, lack of social or parental support, personal or family history of any common mental disorders, being in the second or third trimester, number of births, number of children, number of pregnancies, history of substance use, and history of domestic violence were the factors significantly associated with depressive symptoms (8, 9, 37).

Although antenatal depressive symptoms are a prevalent illness with harmful effects, few studies have been carried out in low-income countries like Ethiopia. A study conducted in 2012 in Southwestern Ethiopia and Addis Ababa city found that the prevalence of depressive symptoms during pregnancy was 19.9% (95% CI, 16.8–23.1) and 24.94% (95% CI: 20.85–29.30%), respectively, using an EPDS cutoff point of 13 and above (9, 38).

Due to the minimal number of studies that have been done in Ethiopia, it is difficult to develop an effective solution for antenatal depressive symptoms. Therefore, the purpose of this study was to ascertain the prevalence of antenatal depressive symptoms and identify associated factors among pregnant women in Northwest Ethiopia.

## Methods and materials

### Study design and study period

An institutional-based cross-sectional study was conducted among pregnant women attending antenatal care services at Comprehensive Specialized Hospitals of Northwest Ethiopia from May to June 2022.

### Study area

The study was conducted in three comprehensive specialized hospitals (CSH) named “the University of Gondar (UOG),” “Tibebe Ghion,” and “Felege Hiwot” Comprehensive Specialized Hospitals (CSH), which are located in the northwest part of Ethiopia.

#### Inclusion criteria

All pregnant women aged 18 and above who visit the antenatal care (ANC) clinics at the University of Gondar (UOG), Tibebe Ghion, and Felege Hiwot CSH have been included.

#### Exclusion criteria

Women who were in labor, severely ill pregnant women, and people who had trouble communicating throughout the data collection time were all excluded from this study.

### Sample size determination

To determine the required sample size, we used a single proportion formula. To calculate the sample size, the following aspects were taken into account: the margin of error (d), which was set at 4%; the level of confidence (95%); the proportion (p), which was set at 24.94% from a previous study that was conducted in Ethiopia (9), and the 10% non-response rate. Therefore, the final sample size was 495.

#### Sampling technique and procedure

After proportional allocation to each institution, we used a systematic random sampling technique to take samples from every other woman who had prenatal care from May to June 2022. After gaining of knowledge of how many pregnant women had visited each hospital in a month since the pandemic began in Ethiopia (650 at UOG, 472 at Tibebe Ghion, and 483 at Felege Hiwot CSHs), the proportional allocation was carried out. The proportional allocation formula resulted in the distribution of 200 pregnant women to UOG, 145 to Tibebe Ghion, and 149 to Felege Hiwot CSHs. Every other pregnant woman was randomly selected, and each hospital's first participant was chosen by lottery method. The facilitator led the chosen pregnant woman to where the data collectors were stationed. A systematic random sample procedure was used for all participants that met the inclusion criteria.

### Data collection tool and procedure

Structured questionnaires that had been validated and pretested were used to collect data from the study participants. Following a 1-day training led by the primary investigator, the data were collected by nine BSc psychiatric professionals.

The structure of the questionnaires consists of seven parts.

#### Demographic characteristics

Participants' demographic information, such as age, ethnicity, religion, education, occupation, marital status, and residency, was collected to obtain baseline data.

#### Obstetrics and gynecological characteristics

The participants and their medical records were used to collect data on gynecological, obstetrical, and associated clinical variables that provided information on gestational age, parity, gravidity, the experience of abortion, type of pregnancy, and other co-morbid illnesses like pregnancy-related hypertension, gestational diabetes mellitus, history of mental illness, and other illnesses.

#### Clinical and related factors

The Edinburgh postnatal depression scale (EPDS), which has 10 items, was used to measure depressive symptoms during pregnancy. The EPDS has been applied in a variety of cultural contexts. The EPDS questionnaire has been validated as effective in measuring antenatal depression (39). Overall, the scores ranged from 0 to 30. In several nations, the screening technique has been proven effective at identifying depression in prenatal and postnatal samples. The tool was validated in public health facilities in Addis Ababa for postpartum and displayed a sensitivity of 84.6%, a specificity of 77.0%, and Cronbach's alpha of 0.71 at the cutoff score of 7/8 (39). Pregnant women typically have higher EDPS cutoff points than postpartum women (34). In this study, a cutoff value of 13 was used to identify pregnant women with depressive symptoms or probable depression, similar to other studies carried out internationally and in Ethiopia (35–37). Women who scored 13 or above during pregnancy were classified as having depressive symptoms (9). The OSLO-3 social support scale was used to evaluate the perception of social support. It comprises a 3-item questionnaire to ascertain the social support status and was evaluated using an OSL-3 validated tool. The Oslo-3 social support scale has a cumulative score range from 3 to 14, and it has three main categories: “poor social support” (scores of 3 to 8), “moderate social support” (9 to 11), and “strong social support” (12 to 14). It has Cronbach's alpha of 0.88, specificity of 67%, and sensitivity of 86% (40).

#### Substance use and related factors

Semi-structured questionnaires adapted from WHO ASSIST V3 were used to measure behavioral aspects such as alcohol, tobacco, khat chewing, and other substance use (31). Pregnant women who used substances such as tobacco, khat, and alcohol only for non-medical purposes during this pregnancy were deemed to be currently using substances, while participants who used substances like these before the current pregnancy were considered to have ever used substances (41).

#### Intimate partner violence characteristics

Intimate partner violence characteristics consist of five questions to assess the prevalence of intimate partner violence. The questionnaire was developed from the WHO's Multi-Country Study on Violence Against Women, and the response to each item was either yes or no. If any questions on the screen were answered yes, the participant was considered positive for intimate partner violence (42).

### Data quality control

Data were collected using face-to-face, structured interviews. The study participants were informed about the general information regarding the study objectives as well as the opportunities or benefits that this study could bring. Finally, the filled-out questionnaires were checked for consistency and completeness daily. High priority was given to the design of data-gathering instruments to ensure data quality. The questionnaire was prepared in English, translated to Amharic (the local language) by fluent speakers of the languages, and back-translated to English by another person to maintain consistency. Information was gathered via questionnaires that were translated into Amharic. The principal investigator trained the data collectors and supervisors on data-gathering techniques for 1 day. 1 week before the actual data collection, the questionnaire was pretested on 5% of the overall sample size, which was excluded from the main survey, and the structure of the questionnaire was amended accordingly.

### Data processing and analysis

Epidata software version 4.6.0.0 was used to code and enter the data, and SPSS version 25 was used to analyze the results. Incomplete questionnaires (*n* = 12) were considered as non-response. By using SPSS version 25, the data were examined to generate descriptive statistics, including means, medians, frequencies, percentages, and standard deviations. The Hosmer–Lemeshow goodness-of-fit was used to verify the fit (*p* = 0.89). Chi-square tests and multicollinearity (VIF values of 1.07 and multicollinearity) assumptions were made. With a 95% confidence interval, adjusted odds ratios were calculated using logistic regression analysis to control for confounding variables. Variables with a *p*-value <0.2 in the bivariate logistic regression analysis were entered into a multivariable logistic regression to detect whether there was a significant association. Outcome and independent variables were entered into the bivariate logistic regression one by one to detect association and multivariable logistic regression to show the presence and strength of association. Statistical significance was determined at a *P*-value of < 0.05 at 95% CI.

### Ethical consideration

Ethical clearance and an official letter of permission were obtained from the Institutional Review Board (IRB) of the University of Gondar and the University of Gondar College of Medicine and health science, respectively. Then, the letters were submitted to UoG, Tibebe Ghion, and Felege Hiwot CSHs, Department of Gynecology and Obstetrics outpatient unit. Before the interview began, all participants received a thorough explanation of the study objectives. Written consent was obtained from each participant before starting data collection. Participants in the study were informed of their right to decline participation. At every stage of data processing, the confidentiality of the information and the privacy of study participants were maintained. Pregnant participants who complained of recent emotional or physical violence, such as experiencing IPV or GBV, and had suicidal thoughts during the time of data collection were advised to visit psychiatry OPDs.

### Patient and public involvement

Pregnant women who attended the ANC clinic at UOG, Tibebe Ghion, and Felege Hiwot CSHs participated in this study. Severely ill pregnant women were not included. Participants were not involved in the study's design or the recruitment process. The result of this study has been submitted to the Department of Psychiatry, UOG, Tibebe Ghion, and Felege Hiwot CSHs.

## Result

### Socio-demographic characteristics

In this study, 473 pregnant women participated with a response rate of 95.55%. The mean age of the respondent was 28.18 years with a standard deviation of SD ± 5.28. The age of the respondents ranges from 18 to 41 years. In total, 436 (92.2%) of the participants were married women, and the majority of the respondents were living in urban areas 420 (88.8%). The majorities of the respondents were Amhara ethnicity 436 (92.2%), and 356 (75.3%) were orthodox Christians (Table 1).

**Table 1:** Socio-demographic characteristics of pregnant women attending antenatal care services at Comprehensive Specialized Hospitals in Northwest Ethiopia, 2022 (n = 473).

**Variables**	**Category**	**Frequency**	**Percentage (%)**
Age	18–24	115	24.3
	25–34	285	60.3
	≥35	73	15.4
Marital status	Married	436	92.2
	Single	22	4.7
	Others^*^	15	3.2
Educational level	No formal education	76	16.1
	Primary school	180	38.1
	Secondary school	99	20.9
	Diploma and above	118	24.9
Occupation	Housewife	168	35.5
	Student	25	5.3
	Self-employed	105	22.2
	Governmental employed	127	26.8
	Unemployed	48	10.1
Religion	Orthodox	356	75.3
	Muslim	60	12.7
	Catholic	37	7.8
	Protestant	17	3.6
	Others^**^	3	0.6
Residence	Urban	420	88.8
	Rural	53	11.2
Ethnicity	Amhara	436	92.2
	Oromo	26	5.5
	Tigre Others^***^	7 4	1.5 0.8

Others^*^: widowed, divorced, separated. Others^**^: Seventh-Day Adventist, and Jehovah's Witness. Others^***^: Wolayta, Benshagul Gumiz, Afar.

### Obstetrical and clinical characteristics of participants

More than one-third of the participants, 186 (39.3), were in the third trimester, and nearly one-third, 133 (28.1%), were experiencing their first pregnancy (primigravida). A total of 336 (71.0%) participants had experienced labor two or more times before, and nearly one-third, 146 (30.9%), of the participants had no child. Sixty-eight (14.4%) women had a history of abortion before. More than 13 out of 100 participants (13.3%) were having wanted pregnancy and nearly 1 out of 10, 38 (8.0%), had chronic medical illnesses such as HIV, COPD, hypertension, and diabetes mellitus, and 35 (7.4%) of the pregnant women had a history of mental illness in their lifetime (Table 2).

**Table 2:** Obstetrical and clinical characteristics of pregnant women attending antenatal care services at Comprehensive Specialized Hospitals in Northwest Ethiopia, 2022 (n = 473).

**Variables**	**Category**	**Frequency**	**Percentage (%)**
Pregnancy term	First trimester	126	26.6
	Second trimester	161	34.0
	Third trimester	186	39.3
Gravidity	Primigravida Multigravida	133 340	28.1 71.9
Parity	Nulliparous	137	29.0
	multiparous	336	71.0
Number of children alive	None	146	30.9
	One	173	36.6
	Two	94	19.9
	Three or more	60	12.7
History of abortion	Yes	68	14.4
	No	405	85.6
Status of pregnancy	Wanted	410	86.7
	Unwanted	63	13.3
History of mental illness	Yes	35	7.4
	No	438	92.6
Chronic medical illness	Yes	38	8.0
	No	435	92.0

### Psychosocial, history of intimate partner violence, and substance use

Out of 100 pregnant women, more than 6 (6.3%) have ever been abused emotionally or physically in their lifetime by someone, and 27 (5.7%) pregnant women have been slapped, kicked, or otherwise physically hurt by someone during the last year. Nineteen (4.0%) pregnant women have been slapped, kicked, or otherwise physically hurt by someone since the current pregnancy. Overall, 68 (14.4%) pregnant women have a history of intimate partner violence.

At least one of the ten (10.4%) pregnant women has a history of using alcohol before the current pregnancy, and 40 (8.5%) were using alcohol since the current pregnancy. Of the participants, 198 (41.9%) pregnant women had poor social support (Table 3).

**Table 3:** Characteristics of psychosocial, history of intimate partner violence, and substance use of pregnant women attending antenatal care at comprehensive and specialized hospitals of Northwest Ethiopia, 2022 (n = 473).

**Variables**	**Category**	**Frequency (%)**	**Percentage (%)**
Ever been emotionally or physically abused	Yes	30	6.3
	No	443	93.7
Within the last year, have you been hit, slapped, kicked, or otherwise physically hurt by someone?	Yes	27	5.7
	No	446	94.3
Since you've been pregnant, have you been slapped, kicked, or otherwise physically hurt by someone?	Yes	19	4.0
	No	454	96.0
Within the past year, has anyone forced you to have sexual activities?	Yes	15	3.2
	No	458	96.8
Are you afraid of your partner or anyone you listed above?	Yes	21	4.4
	No	452	95.6
Intimate partner violence	Yes	68	14.4
	No	405	85.6
Ever use of alcohol	Yes	49	10.4
	No	424	89.6
Current use of alcohol	Yes	40	8.5
	No	433	91.5
Lifetime cigarette smoking	Yes	2	0.4
	No	471	99.6
Social support	Poor	198	41.9
	Moderate	151	31.9
	Strong	124	26.2

#### Prevalence of antenatal depression

The prevalence of depressive symptoms among pregnant women is 19.2% (95% CI: 15.8, 23.1).

### Factors associated with antenatal depression

Marital status, educational level, residency, status of pregnancy, gestational age, having chronic disease, use of alcohol, status of social support, and intimate partner violence were variables that had a *p*-value of <0.2 at bivariate logistic regression analysis. Of those living in rural areas, being in the first and third trimesters of pregnancy, use of alcohol before getting pregnant, having moderate to poor social support, and having a history of intimate partner violence were found to be significantly associated with depression in the multivariable logistic regression analysis at a *p*-value of <0.05.

The magnitude of depressive symptoms among the participants was found to be 2.5 times more likely among pregnant women living in rural areas as compared to pregnant women living in urban areas (AOR = 2.58, 95% CI: 1.267, 5.256). The odds of having antenatal depression among participants were 4.4 and 5.4 times higher among second- and third-trimester pregnant women as compared to first-trimester pregnant women [(AOR= 4.40, 95% CI: 1.949, 9.966) and (AOR= 5.42, 95% CI: 2.438, 12.028), respectively].

Pregnant women who have a history of alcohol use are more than two times more likely to have antenatal depression as compared with pregnant women who have never used alcohol (AOR=2.41, 95% CI: 1.099, 5.260). The probability of having depressive symptoms was more than 2.5 and 2.4 times higher among pregnant women who have moderate and poor social support as compared to pregnant women who have strong social support [(AOR= 2.55, 95% CI: 1.220, 5.338) and (AOR= 2.41, 95% CI: 1.106, 5.268), respectively]. The odds of having depressive symptoms among pregnant women who had intimate partner violence were 2.6 times more likely as compared with pregnant women who had not had such exposure (AOR = 2.67, 95% CI: 1.416, 5.016) (Table 4).

**Table 4:** Bivariate and multiple logistic regression analyses of factors significantly associated with antenatal depression among pregnant women attending antenatal care services at Comprehensive Specialized Hospitals in Northwest Ethiopia, 2022 (n = 473).

**Variables**	**Depressive symptom**		**COR 95% CI**	**AOR 95% CI**	***P*-value**
**Yes**	**No**
**Marital status**					
Married	103	333	1	1
Single	13	9	3.23 (1.332, 7.819)^**^	1.96 (0.695, 5.539)	0.203
Others^*^	10	5	2.33 (0.775, 7.013)	1.98 (0.566, 6.950)	0.285
**Educational level**					
No formal education	39	37	2.12 (1.043, 4.316)^*^	0.93 (0.398, 2.174)	0.866
Primary school	134	46	1.44 (0.774, 2.668)	1.13 (0.573, 2.216)	0.730
Secondary school	22	77	0.98 (0.992, 2.088)	0.89 (0.397, 2.014)	0.787
Diploma and above	19	99	1	1
**Residency**					
Urban	89	331	1	1
Rural	37	16	3.28 (1.788, 6.023)^**^	2.58 (1.267, 5.256)	0.009
**Status of pregnancy**					
Wanted	101	309	1	1
Unwanted	25	3	1.85 (1.011, 3.373)^*^	1.73 (0.860, 3.477)	0.124
**Gestational age**					
First trimester	25	101	1	1
Second trimester	49	112	3.22 (1.527, 6.799)^*^	4.40 (1.949, 9.966)	0.000
Third trimester	52	134	3.81 (1.843, 7.884)^**^	5.42 (2.438, 12.028)	0.000
**Having chronic disease**					
Yes	101	334	2.71 (1.342, 5.481)^*^	0.58 (0.260, 1.320)	0.197
No	25	13	1	1
**Alcohol us**					
Yes	12	36	1.602 (0.811, 3.162)	2.41 (1.099, 5.260)	0.028
No	78	346	1
**Social support**					
Strong	16	108	1	1
Moderate	52	99	3.02 (1.496, 6.100)^*^	2.55 (1.220, 5.338)	0.013
Poor	58	108	3.10 (1.501, 6.402)^*^	2.41 (1.106, 5.268)	0.027
**Intimate partner violence**					
Yes	44	24	2.98 (1.707, 5.223)^*^	2.67 (1.416, 5.016)	0.002
No	82	323	1	1

* *p*-value < 0.05,

** *p*-value < 0.001. COR, crude odd ratio. AOR → adjusted odd ratio. 1 for reference. others^*^: widowed, divorced, separated.

## Discussion

Overall, this study reveals that the prevalence of depressive symptoms among pregnant women attending antenatal care at comprehensive and specialized hospitals in northwest Ethiopia was 19.2% (95% CI: 15.8, 23.1). This study was in line with studies conducted in Ethiopia (17.8%, 17.9%, 19.9%, and 23%), Brazil (20.4%), Bangladesh (18%), India (21% and 22%), China (16.3%), and a systematic review and meta-analysis study conducted globally (20.7%) (5, 19, 29, 43–49).

The prevalence of this study was higher than studies conducted in Brazil (14.8%), Avon, Ohio (13.5%), New Zealand (8.5%), and a systematic review study conducted globally (7.4%) (10, 11, 37, 45). The possible reasons for this discrepancy might be the population socio-demographic difference (10, 11, 37), the use of different study designs (some of the previous studies used other study designs such as longitudinal and systematic review study designs) (11, 45), the difference in study duration, for instance, a few studies were conducted 18 years ago (10), and the use of different measurement tools other than EPDS such as the Hospital Anxiety and Depression Scale (HADS) (10, 37). Some of the studies were conducted at the community level (11, 37), and the others had a small sample size (37); those discrepancies might have contributed to the difference.

The magnitude of this study was lower than studies conducted in Ethiopia (24.49%−34.5%) (2, 9, 50–52). The possible explanation for this discrepancy could be sampling techniques. Some of the previous studies used consecutive sampling techniques (9), whereas in this study we used a random sampling technique. A study period (9), a small number of participants (2, 51), and the use of different measurement tools for the outcome such as “Beck Depression Inventory” which has higher sensitivity as compared with EPDS (51, 53) might be the contributors for this discrepancy. The finding of this study is also lower than studies conducted in South Africa (25% and 39%) (20, 54), Jamaica (56%) (55), Pakistan (25%) (22), Montreal, Canada among immigrant pregnant women (42%%) (1), and Britain (36.1%−43.3%) (56, 57). The possible reasons for these variations might be the study period (55), socio-cultural characteristics of the participants (1, 20, 22, 56), and the use of different measurement tools for the outcome; moreover, a study conducted in Jamaica used “the Zung Self-Rating Depression Scale” (55) and study design (20, 54), which might also have a contribution.

Factors such as living in a rural area, the second and third trimesters of pregnancy, the use of alcohol, having poor or moderate social support, and having a history of intimate partner violence were found to be significantly associated with depressive symptoms during pregnancy.

Living in a rural area has a significant association (*p* < 0.009) with depressive symptoms during pregnancy. Living in a rural area has a significant association (*p* < 0.009) with depressive symptoms during pregnancy. The likelihood of experiencing depressive symptoms among pregnant women who live in rural areas was more than 2.5 times higher than among pregnant women who live in urban areas. This finding was supported by studies conducted in the Democratic Republic of the Congo (58) and China (46). The possible reason might be educational attainment and income may be lower in rural areas than in urban areas (59, 60), and lower educational attainment and lower income are strongly associated with depressive symptoms (61–63). Additionally, low population density may be more socially isolating for people living in rural and remote areas, and this might increase the chance to have depressive symptoms (63).

Second- and third-trimester pregnancy terms were seen as contributing factors for experiencing depressive symptoms. Respondents who were in their second and third trimesters had the odds of having depressive symptoms more than four and five times, respectively, as compared with pregnant women in their first trimester. This finding was supported by studies conducted at Maichew and Jimma in Ethiopia (51, 64), China (65), and a systematic review study conducted globally (10). The possible reasons for this association might be due to increased body weight, stressful feelings, and other somatic symptoms as the pregnancy progresses, which might cause emotional and mental distress that could increase the risk of developing depressive symptoms (35, 54, 63). Third-trimester pregnancy is associated with trouble with sleeping, shortness of breath, and false labor contractions which might account for depression (66). These pregnancy-related changes can precipitate depression and resemble vegetative symptoms of depression (64, 67). In addition, as the stage of pregnancy progress, there are also gradual increases in hormone concentrations within the cortisol stress system—the hypothalamic–pituitary–adrenal (HPA) axis overactivity, which has been found in people with depression (13, 68).

Those pregnant women who had been using alcohol before their current pregnancy were more than two times more likely to have depressive symptoms as compared to pregnant women who did not use alcohol. The finding of this study is supported by studies conducted in South India (44), USA (69), and Brazil (37). The possible reason for this association might be that the presence of mental disorders may contribute to the use of alcohol or vice versa (70). The correlation found in this study enables us to deduce that pregnant women who drink tend to experience greater depressive symptoms, while it is also possible that depression occurs before alcohol consumption, in which case pregnant women drink to lessen the symptoms of depression (37, 69). Alcohol is a depressant that slows down brain activity as well as causes depression due to neurochemical, physical, emotional, or psychological disturbances (71, 72).

The result of this study showed that pregnant women who have strong social support were less likely to have depressive symptoms during pregnancy as compared with pregnant women who have moderate or poor social support. When compared to pregnant women with strong social support, depressive symptoms were more than two times more likely to occur in those with poor and moderate social support. This finding is supported by studies conducted in Canada, a systematic review of reviews (an umbrella review) (23), and Northeast Ethiopia (48). This could be explained by the fact that women who receive social support from their partners, families, and friends throughout pregnancy may find it easier to deal with difficult life circumstances (51, 64).

The likelihood of having depressive symptoms among pregnant women who had intimate partner violence during their lifetime was 2.6 times higher as compared to pregnant women without such exposure. This finding was consistent with other studies such as systematic reviews and meta-analyses (50), rural Bangladesh and King's College London (13, 29), Brazil (37), South India (44), Cape Town in South Africa (20), and Northeast Ethiopia (48). The association between these variables could be explained by the accumulation of stress triggered by intimate partner violence, which is fundamentally degrading, particularly during the reproductive years when escape options are sometimes limited (13, 68). In light of the social theory of the origin of this condition put forth by Brown and Harris in 1978 (37, 73), which contends that depression is a result of an individual's experience of being humiliated and imprisoned, this humiliation may cause the start of depression (13). In light of this, the results of the current study may be justified on the grounds that pregnant women who have experienced abuse may experience sadness and pain when they think back on the humiliation they endured (1, 33).

## Limitation of study

Despite the fact that this study made significant discoveries in the understudied areas of depressive symptoms in pregnant women in Ethiopia, several limitations should be considered when generalizing the findings. The first limitation is the potential for underreporting of the events, which may be due to the way we collected the data. Due to the use of a face-to-face interviewing method, there may be some recall bias. Individuals who had no symptoms of depression may have less motivation to recall earlier exposure than individuals with the symptoms.

Second, due to the nature of the cross-sectional study design, it is not possible to report the temporal cause-and-effect relationship, such as the relationship between depressive symptoms and alcohol use.

## Conclusion and recommendation

The prevalence of depressive symptoms among pregnant women was high. Living in rural areas, second and third trimesters, use of alcohol, having moderate to poor social support, and having a history of intimate partner violence were variables significantly associated with experiencing depressive symptoms during pregnancy.

This study recommends that all pregnant women should be screened for depressive symptoms and those screened positive should be referred for treatment during the second and third trimesters.

Special attention should be given to pregnant women who reside in rural areas, have a history of drinking alcohol before getting pregnant, have moderate or poor social support, and have been victims of intimate partner violence.

## Data availability statement

The original contributions presented in the study are included in the article/supplementary material, further inquiries can be directed to the corresponding author.

## Ethics statement

The studies involving human participants were reviewed and approved by Institutional Review Board (IRB) of University of Gondar. The patients/participants provided their written informed consent to participate in this study.

## Author contributions

GT wrote the proposal, participated in data collection, analyzed the data, and drafted the manuscript. GT, GN, GR, and MM were involved in the designing of the proposal, data collection, and analysis. MM participated in data analysis. All authors read and approved the final manuscript.
